# Mitotic polarization of transcription factors during asymmetric division establishes fate of forming cancer cells

**DOI:** 10.1038/s41467-018-04663-1

**Published:** 2018-06-21

**Authors:** Yongqing Liu, Laura Siles, Xiaoqin Lu, Kevin C. Dean, Miriam Cuatrecasas, Antonio Postigo, Douglas C. Dean

**Affiliations:** 1 Molecular Targets Program, James Brown Cancer Center, University of Louisville Health Sciences Center, Louisville, KY 40202 USA; 2 Department of Ophthalmology and Visual Sciences, University of Louisville Health Sciences Center, Louisville, KY 40202 USA; 3 Birth Defects Center, University of Louisville Health Sciences Center, Louisville, KY 40202 USA; 4 Group of Transcriptional Regulation of Gene Expression, Institut d’Investigacions Biomèdiques August Pi i Sunyer (IDIBAPS), CIBERehd, 08010 Barcelona, Spain; 5 Department of Pathology, Centro de Diagnóstico Biomédico (CDB) Hospital Clínic, University of Barcelona, 08010 Barcelona, Spain; 6 Tumor Bank-Biobanc, IDIBAPS, 08010 Barcelona, Spain; 7 ICREA, 08010 Barcelona, Spain

## Abstract

A model of K-Ras-initiated lung cancer was used to follow the transition of precancerous adenoma to adenocarcinoma. In hypoxic, Tgf-β1-rich interiors of adenomas, we show that adenoma cells divide asymmetrically to produce cancer-generating cells highlighted by epithelial mesenchymal transition and a CD44/Zeb1 loop. In these cells, Zeb1 represses the Smad inhibitor Zeb2/Sip1, causing Pten loss and launching Tgf-β1 signaling that drives nuclear translocation of Yap1. Surprisingly, the nuclear polarization of transcription factors during mitosis establishes parent and daughter fates prior to cytokinesis in sequential asymmetric divisions that generate cancer cells from precancerous lesions. Mutation or knockdown of *Zeb1* in the lung blocked the production of CD44^hi^, Zeb1^hi^ cancer-generating cells from adenoma cells. A CD44/Zeb1 loop then initiates two-step transition of precancerous cells to cancer cells via a stable intermediate population of cancer-generating cells. We show these initial cancer-generating cells are independent of cancer stem cells generated in tumors by p53-regulated reprogramming of existing cancer cells.

## Abstract (web summary)

Transition from premalignant lesion to cancer cell highlights tumor initiation. Here, the authors use a model of K-Ras-initiated lung cancer to document two successive asymmetric divisions, each driven by mitotic polarization of key transcription factors, which lead to generation of initial cancer cells.

## Introduction

A small population of cells, termed cancer stem cells (CSC) or tumor-initiating cells, have been identified in many tumors, including lung adenocarcinoma (AC)^1–4^. These cells can divide asymmetrically to generate cancer cells, while maintaining their numbers in the tumor. CSC were thought to arise from the transformation of adult stem cells or progenitor population persisting in tissues, and these cells, in turn, were responsible for the generation of initial cancer cells. But, recent studies demonstrate that existing cancer cells undergo reversible reprogramming to generate CSC, which are then thought to be critical for maintaining cancer cell numbers in tumors and generating new cancer cells following therapy^1–3^. Thus, a relationship between CSC generated from reprogramming of existing cancer cells and the pathway leading to initial cancer cell generation are still being unraveled.

Although CSC display normal stem cell properties such as asymmetric division, there are key differences in pathways and gene expression patterns in CSC vs. stem cells. Perhaps, the foremost among these differences is tissue stems cells display an epithelial-like phenotype, and iduced pluripotent stem cells (iPS) reprogramming to generate stem-like cells requires a mesenchymal-to-epithelial transition^4^, whereas CSC are characterized by an opposing epithelial mesenchymal transition (EMT), which can be driven by induction of EMT transcription factors such as Zeb1^2,5^. This EMT in CSC is linked to high expression of CD44, which marks CSC in tumors including breast and lung cancers^6–9^, and a positive CD44/Zeb1 loop has been shown to drive EMT and reprogramming of existing cancer cells to a CSC phenotype^10,11^. This loop can be initiated by Tgf-β induction of Zeb1 in cell culture^2^, but it is unclear if such a loop is present or functional in vivo.

We utilized a K-Ras-initiated model of lung AC^12^ to search for a CD44/Zeb1 loop in vivo, and address its potential role in cancer cell generation. Ras pathway mutations, including K-Ras itself and EGFR, have been widely utilized in mouse models of human lung AC^13^. These mutations are mutually exclusive in human lung AC, suggesting that they are redundant and thus equivalent in Ras pathway activation in the lung^14^. Mutations such as *Pten* or *p53* affect tumor progression in this K-Ras model, and they have been widely utilized with K-Ras to evaluate their roles in tumors. Notably, *Pten* is not mutated in K-Ras-initiated tumors such as lung and pancreatic AC, but instead, its expression is somehow repressed as these tumors progress, accounting for *Pten* mutation accelerating tumor progression in these mice^15–17^. Compound mutation of *p53* does not affect cancer cell generation or their expansion into tumors^18,19^. Instead, its mutation allows K-Ras-initiated tumors to transition to metastasis, implying p53 is acting later to promote cancer cell metastasis in this model.

**Fig. 1:**
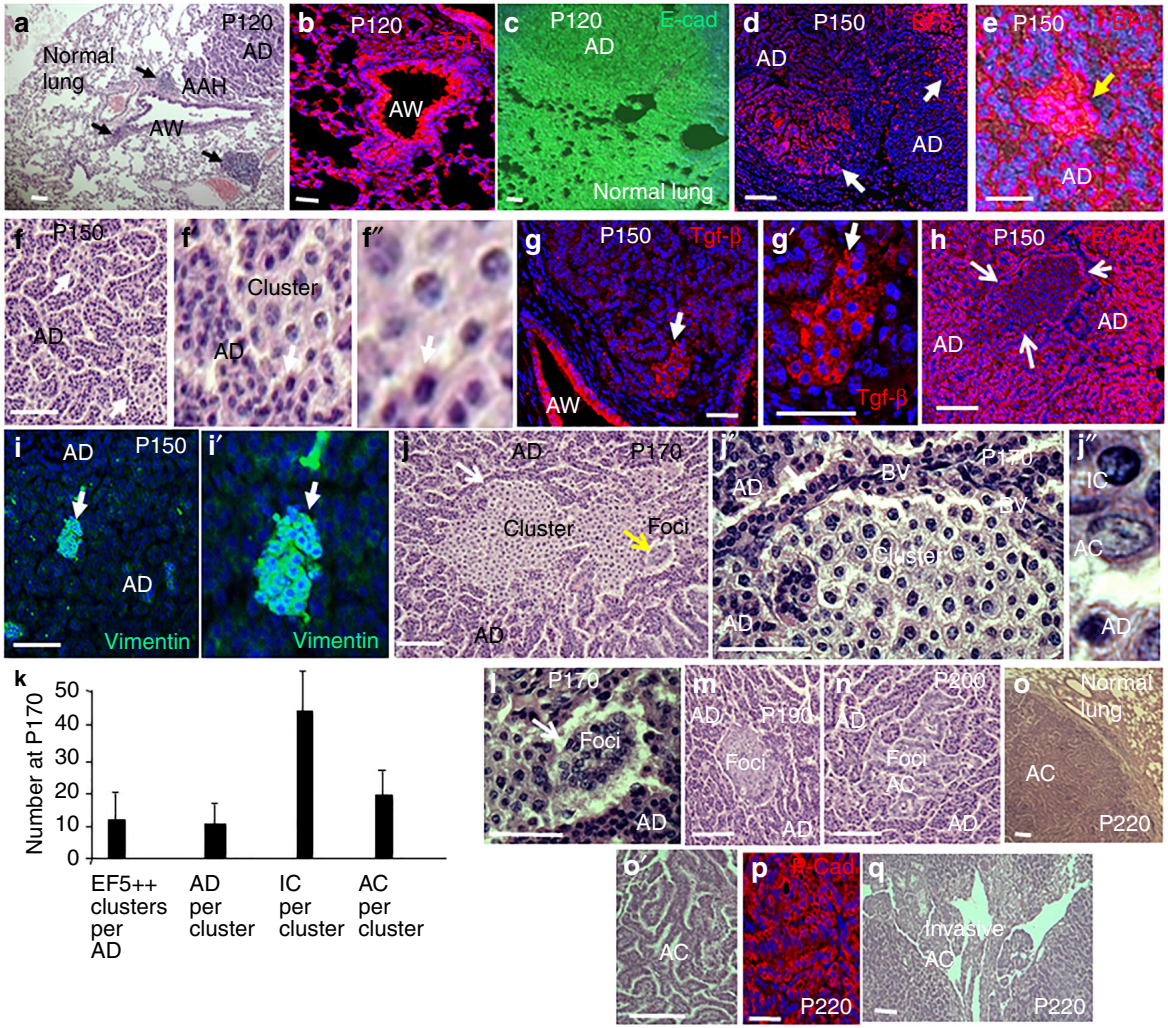
Inflammation, Tgf-β1 accumulation, hypoxia, and EMT mark cancer cell-generating clusters in expanding adenomas. **a** H&E staining showing sites of atypical adenomatous hyperplasia (AAH) originate around bronchial airways (AW), and then begin expanding into precancerous adenomas (AD) by P120 in K-Ras mutant mice. These sites are linked to infiltrating inflammatory cells. High concentrations of inflammatory cells are shown by black arrows, but lower levels of these infiltrating cells have spread throughout the lung. **b** Sites of AAH, AD, and inflammation are rich in Tgf-β1. **c** Normal lung, AAH. and AD express E-cadherin (E-cad). **d** Immunostaining for EF5 shows that by P150 the interiors of expanding AD are hypoxic (EF5+). **e** Highly EF5-positive sites (EF5++) are present in the hypoxic interiors of expanding AD. **f**–**f″** EF5++ sites represent small clusters of cells with decondensed chromatin. Arrows in panel **f** show two such clusters surrounded by AD cells, which contain small nuclei with dense heterochromatin. Arrows in panels **f′** and **f″** show the same location. **g**, **g′** EF5++ clusters are sites of Tgf-β accumulation. Arrows show the same position in panels **g** and **g′**. **h** Consistent with EMT, loss of E-cad marks cell clusters. Arrows show the outline of the cluster. **i**, **i′** The mesenchymal marker Vimentin is induced in cell clusters. Arrows show the same position in panels **i** and **i′**. **j**–**j″** By P170, clusters have expanded. The yellow arrow in panel **j** shows cancer cells forming dense foci (see panel **k**). The white arrows in panels **j** and **j′** show the same position. “BV” are blood vessels within AD at the AD/cluster border. Note absence of BV within the cluster. Panel **j″** shows an AD cell with a small heterochromatin dense nucleus, a euchromatic AC cell containing a larger euchromatic vesicular nucleus with an irregular contour and nucleolar enlargement, and a cell with an intermediate nuclear morphology evidenced by heterochromatin decondensation. These cells displaying an intermediate nuclear morphology are termed intermediate cells (IC). **k** Hypoxic EF5++ clusters, first identified in panel **e**, were counted per adenoma, and cell types were counted within clusters at P170 in four lungs (20 adenomas). **l**–**n** AC cells assemble into dense foci and form papillary structures. The yellow arrow in panel **l** shows the same position in panel **j**. **o**, **o′** By P220, AC cells had formed large tumors. **p** As opposed to IC, but similar to adenoma cells, the tumors cells expressed E-cad and thus did not show evidence of EMT. **q** Invasion of expanding tumors into airways is shown. *n* > 5 for each age. Bars are 50 μm

As opposed to compound mutations generated simultaneously in mouse models, mutations are thought to arise sequentially over a long period in patients. In this regard, it is of note that K-Ras mutation alone initiates a pathway leading to lung AC in mice, but with this single mutation, the process is highlighted by a protracted period of precancerous lesion expansion^12,20^. In these mice, precancerous subpleural adenomas form around bronchial airways (Fig. 1a). AC cells appear later in these adenomas, and they expand into large tumors that invade airways.

Elegant lineage mapping in the lung has shown both Clara and ATII cells are the targets of K-Ras transformation responsible for precancerous adenoma generation^21,22^. In the absence of concurrent *p53* mutation, transformed ATII cells are the only lineage that transitions to AC, thus AC in this model are derived from ATII cells. But, as noted above, steps involved in the transition of ATII-derived adenoma cells to AC are still being unraveled. We focused our studies on such sites of initial cancer cell generation within expanding adenomas in the lungs of mice expressing mutant K-Ras. We found an evidence that a positive CD44/Zeb1 loop is initiated in small clusters of cells derived from asymmetrically dividing adenoma cells at sites of hypoxia, EMT, and Tgf-β1 accumulation in the interiors of expanding adenomas. Zeb1 repression of the Smad inhibitor Zeb2/Sip1 launches Tgf-β1 signaling in these cell clusters, and they activate transcription factors and pathways known to be important for CSC formation. We show these adenoma-derived cluster cells are initial cancer-generating cells (CGC), and they, in turn, divide asymmetrically to produce AC cells. These two sequential asymmetric divisions that generate cancer cells from precancerous lesions both show early nuclear polarization of key transcription factors during mitosis, and resulting asymmetric expression of their target genes in opposite cell poles then establishes parental and daughter fates prior to cytokinesis. Mutation or knockdown of *Zeb1* in the lung blocked the production of these Zeb1^hi^, CD44^hi^ cancer-generating cluster cells from adenoma cells, in turn inhibiting cancer cell formation. These results provide evidence that a CD44/Zeb1 loop is activated in vivo at sites of hypoxia and Tgf-β1 accumulation in expanding precancerous adenomas, and this loop then initiates a two-step pathway in which precancerous cells transition to cancer cells via a stable intermediate population of CGC. We provide evidence that this population of initial CGC is independent of CSC that are generated in tumors via p53-regulated reprogramming of existing cancer cells.

## Results

### An Intermediate Population Arises in the Hypoxic Interior of Precancerous Adenomas

In response to K-Ras mutation in the mouse lung, atypical adenomatous hyperplasia (AAH) develops around bronchial airways, and these cells expanded to form benign subpleural precancerous adenomas by postnatal day (P) 120^12,20^ (Fig. 1a). Inflammatory infiltrate and Tgf-β1 accumulation was evident at these sites of AAH and adenoma outgrowth (Fig. 1a, b). Although Tgf-β is a known driver of EMT transcription factor expression and thus EMT, cells in AAH and adenomas, as well as adjacent normal lung epithelium, continued to express the epithelial specification protein E-cadherin, and they failed to induce the mesenchymal marker Vimentin, indicating they had not undergone EMT (Fig. 1c). By P150, the interior of expanding adenomas showed evidence of hypoxia, detected by immunostaining for EF5 (EF5+) (Fig. 1d). Within these regions, small sites of intense EF5 immunostaining (EF5++) appeared (Fig. 1e). These sites consisted of cell clusters at sites of Tgf-β1 accumulation that had undergone EMT, as evidenced by the loss of E-cadherin and induction of Vimentin (Fig. 1f–i’). These cluster cells displayed the decondensation of nuclear heterochromatin, and in this way, were also distinct from surrounding adenoma cells, which contained small nuclei with densely packed chromatin (Fig. 1f–f”). By P170, the clusters had expanded, and although blood vessels were evident in adenoma at the border of the clusters, these hypoxic clusters themselves were devoid of vessels (Fig. 1j, j’). At this age, cancer cells, characterized by large euchromatic vesicular nuclei with an irregular contour and nuclear enlargement, were scattered through the clusters (Fig. 1j–j”). The clusters at P170 then contained cells with three different nuclear morphologies: AD cells with small nuclei containing densely packed heterochromatin, cancer cells with large euchromatic nuclei, and intermediate cells (IC) with nuclear size and heterochromatin content between adenoma and cancer cells (Fig. 1j”). Clusters per adenoma are quantified in Fig. 1k, and IC are the predominant cells within these clusters at P170 (Fig. 1k). As the number of cancer cells increased, they formed dense foci and began to display papillary structure (Fig. 1j, l–n). As opposed to the E-cadherin− IC, the cancer cells expressed E-cadherin and thus did not show evidence of EMT, and by P220, they had expanded into large tumors that invaded into airways (Fig. 1o–q). Beyond nuclear morphology, the expression of IC also distinguishes them in both adenoma and cancer cells.

**Fig. 2:**
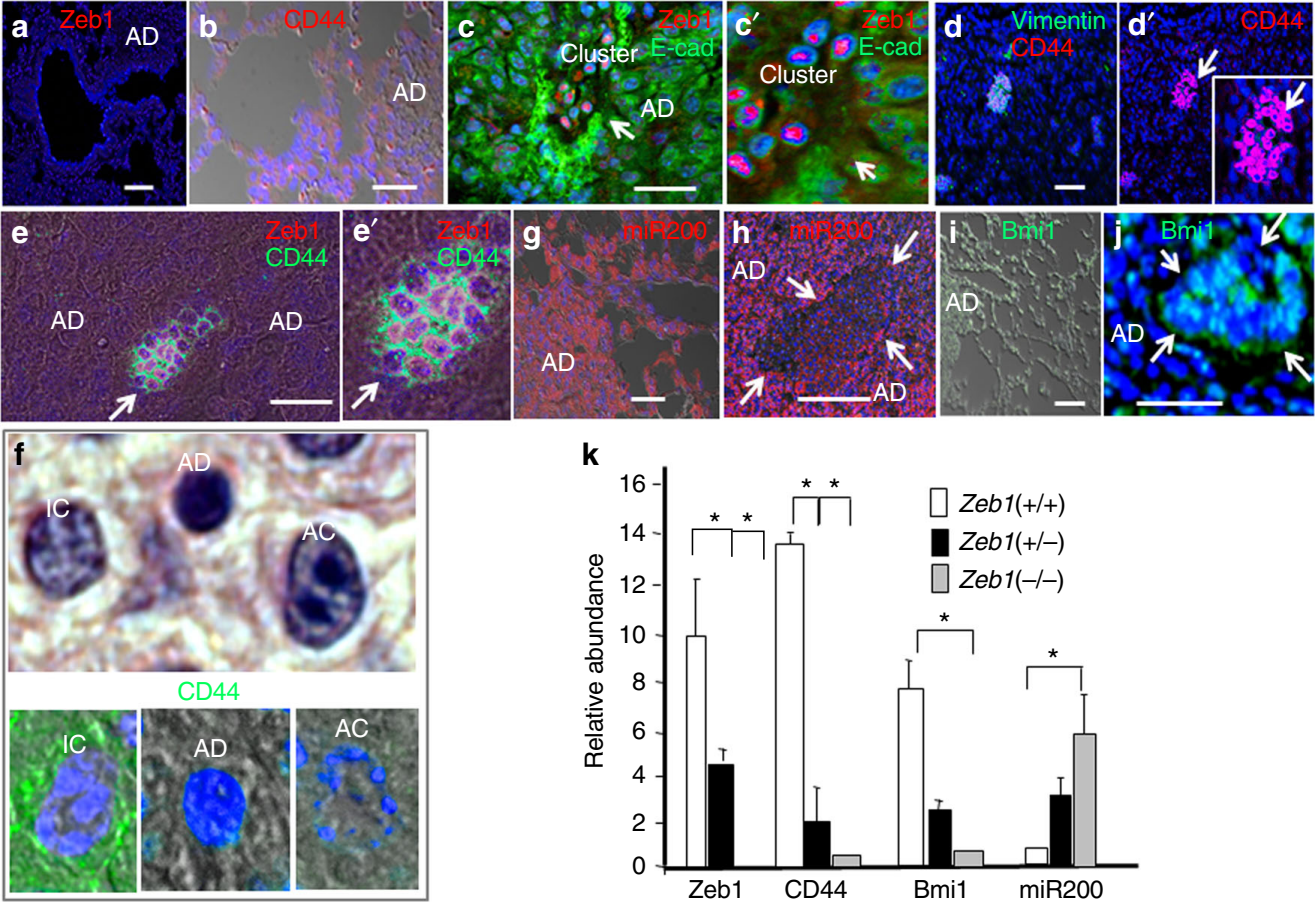
A CD44/Zeb1 loop marks IC. **a** Expression of Zeb1 is low in normal lung, AAH, and AD at P170. **b** CD44 is likewise low in normal lung, AAH, and AD at P170. **c**, **c′** Zeb1 is induced and E-cad is repressed in cell clusters. **d**, **d′** CD44 and Vimentin are induced along with Zeb1 in cell clusters. Arrows indicate the same position. **e**, **e′** Zeb1 and CD44 are co-expressed in cell clusters. Arrows indicate the same position. **f** In cell clusters, CD44 is expressed on IC but not AD or cancer cells (AC). **g** In situ hybridization showing miR-200 is low in normal lung, AAH, and AD. **h** miR-200 is repressed in clusters. Arrows show the border of the cluster. **i** Bmi1 is low in normal lung, AAH, and AD. **j** Bmi1 is induced in clusters. **k** Real-time PCR showing heterozygous mutation of *Zeb1* in mouse embryo fibroblasts (MEFs) is sufficient for induction of miR-200, and repression of CD44 and Bmi1 mRNAs in cell culture. Bars are 50 μm

Consistent with their lack of E-cadherin and expression of Vimentin, both characteristics of EMT, Zeb1, and CD44 expression were first observed in these hypoxic cell clusters exclusively on IC (Fig. 2a–f). It has been demonstrated that Zeb1 represses transcription of the miR-200 family, which has been shown to target mRNAs including CD44 and the CSC transcription factor Bmi1^23–28^. We reasoned that the transcriptional repressor Zeb1, might be important for CD44 expression via it repression of miR-200. In situ hybridization showed that miR-200 is expressed in AAH, adenomas, and surrounding normal lung (Fig. 2g), but it was repressed with Zeb1 induction in clusters (Fig. 2h). As with CD44, Bmi1 was also induced with miR-200 repression in clusters (Fig. 2i, j). Consistently, in cell culture heterozygous mutation of *Zeb1* in primary cultures of mouse embryo fibroblasts (MEFs), to reduce its level, or Zeb1 knockdown was sufficient for induction of miR-200 and repression of CD44 and Bmi1 mRNAs (Fig. 2k).

### Zeb1 Repression of Zeb2 Removes a Block in Tgf-β1 Signaling

Tgf-β classically induces Zeb1 to repress epithelial specification genes such as E-cadherin during EMT^25,29^, implying Tgf-β1 accumulating at sites of tumor formation might be important for driving EMT. Other EMT transcription factors, such as Snail, are also induced by Tgf-β, but they rapidly decline and only Zeb1 maintains repression of epithelial genes during EMT induced by Tgf-β^30^. Beyond repression of epithelial genes, mesenchymal genes must be induced for full EMT, and mesenchymal genes, such as Vimentin, are directly activated by Smads in response to Tgf-β, demonstrating roles for Tgf-β signaling in both Zeb1-mediated repression of epithelial genes and direct induction of mesenchymal genes in EMT. Despite the widespread accumulation of Tgf-β1, AAH, adenoma, and surrounding normal lung continued to display an epithelial expression pattern (Fig. 1c), implying Tgf-β1 signaling might somehow be blocked in these cells, restricting Tgf-β1-driven EMT to cells in clusters.

*Zeb1* and *Zeb2* are related genes containing a common repressor domain^31^, leading to the notion that Zeb2 functionally overlaps with Zeb1 in cancer. However, Zeb2 contains a Smad-binding domain that blocks R-Smad activity, and it was first identified as Smad-interacting protein1 (Sip1)^32^. A switch from Zeb2 to Zeb1 highlights malignant transition in melanoma, and Zeb1 acted as an oncogene, whereas Zeb2 was a tumor suppressor in xenographic transplants of melanoma cell lines^33–35^. These results highlight opposing expression patterns and suggest different roles for Zeb1 and Zeb2 in melanoma, but such a transition from Zeb2 to Zeb1 has not been evaluated in lung cancer, and it is unclear if any functional differences in melanoma might be linked to Zeb2-mediated Smad inhibition. Nevertheless, mutation of *Zeb2* revealed it has a critical role in the inhibition of Tgf-β family signaling to prevent competing mesoderm differentiation during neuroectoderm formation^36,37^, demonstrating its Smad inhibition is important in vivo during development.

**Fig. 3:**
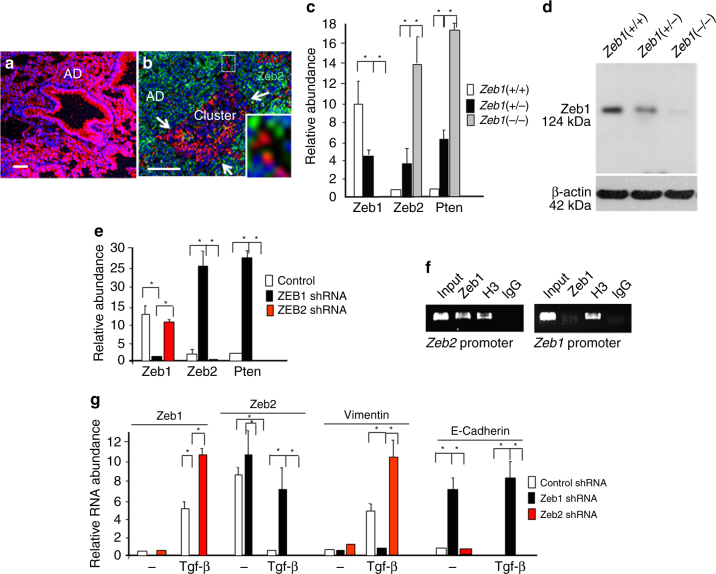
Zeb1 represses Zeb2 to launch Tgf-β1 signaling capacity. **a** Immunostaining showing Zeb2 is high in normal lung, AAH, and AD. **b** Zeb2 is repressed as Zeb1 is induced in clusters at P170. **c** Real-time PCR showing mutation of *Zeb1* in cell culture leads to induction of Zeb2 and Pten mRNAs. **d** Western blot showing that Zeb1 protein expression parallels mRNA levels with *Zeb1* mutation. **e** Lentivirus shRNA knockdown of Zeb1 leads to induction of Zeb2 mRNA, and knockdown of Zeb2 causes loss of Pten mRNA in cell culture. **f** Chromatin immunoprecipitation analysis showing Zeb1 is bound to the *Zeb2* promoter in vivo. **g** The level of Zeb2 accumulation in the absence of Zeb1 is sufficient to block Tgf-β1 signaling in cell culture. Cells were treated with 75 pM Tgf-β1 for 12 h. “Control” indicates cells were infected with a lentivirus containing a scrambled shRNA sequence (Methods). Bars are 50 μm

As opposed to Zeb1, we found immunostaining for Zeb2 in AAH, adenomas, and normal lung, and it was downregulated with Zeb1 induction in hypoxic cell clusters forming in the interior of adenomas (Fig. 3a, b). These results demonstrate a Zeb2-to-Zeb1 switch linked to EMT, and they raised the possibilities, first that elevated Zeb2 might be responsible for blocking Tgf-β1 signaling in normal epithelium, AAH, and adenomas, and thereby preventing EMT, and, second Zeb1 repression of Zeb2 might be responsible for their switch in expression.

We turned to cell culture to examine these possibilities. Knockdown or mutation of *Zeb1* led to the induction of Zeb2 mRNA (Fig. 3c–e). But, knockdown of Zeb2 did not affect the level of Zeb1 mRNA. Consistent with Zeb1 directly repressing Zeb2, we found Zeb1 bound to the Zeb2 promoter in vivo using chromatin immunoprecipitation assays, but Zeb2 was not detected at the Zeb1 promoter in the cells (Fig. 3f). We concluded that Zeb1 is directly repressing *Zeb2*.

Knockdown of Zeb1 led to the de-repression of E-cadherin mRNA, and Tgf-β1 was then unable to repress its expression (Fig. 3g). Tgf-β1 induction of Vimentin mRNA was also blocked by Zeb1 knockdown. By contrast, knockdown of Zeb2 augmented Tgf-β1 induction of Zeb1 and Vimentin mRNAs. We concluded that the level of Zeb2 accumulating in the absence of Zeb1 is sufficient to block Tgf-β1 signaling, and thus Zeb1 repression of Zeb2 is acting to relieve the inhibition of Tgf-β1 signaling.

### Zeb2 Smad Inhibition Blocks Yap1 Nuclear Translocation

Yap1 is a key target in regulating cell–cell contact inhibition^26,38,39^. In this capacity, it is required during the development to block progenitor differentiation allowing outgrowth of forming organs, and in cancer, it is oncogenic, critical for CSC generation and it transitions tumor cells to Ras pathway independence^40–42^. Activation of the Hippo pathway, for example, in response to cell–cell contact, results in Rassf1a binding to Yap1, blocking its interaction with Smad co-transcription factors that mediates Yap1 nuclear translocation and function^43^. In response to Tgf-β signaling, Rassf1a is degraded by Itch and it has been shown this is sufficient for Yap1 association with Smads and nuclear translocation of a functional Yap1–Smad transactivation complex.

**Fig. 4:**
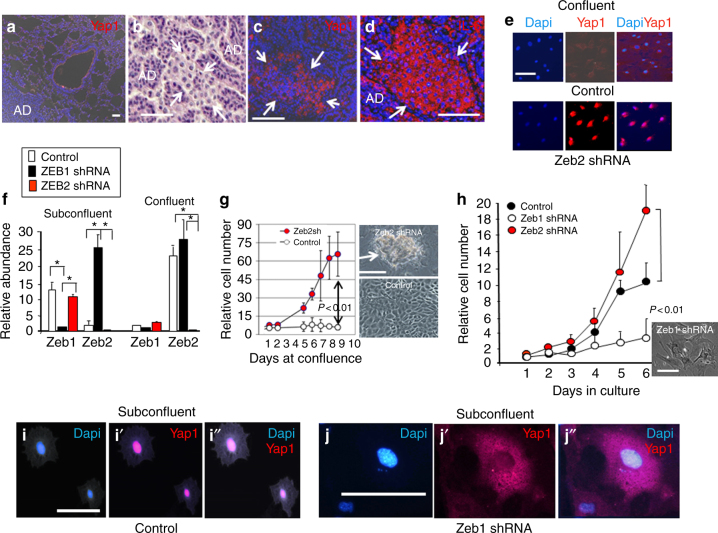
Zeb2 blocks nuclear translocation of Yap1. **a** Immunostaining showing Yap1 is not concentrated in the nucleus in normal lung, AAH, and AD. **b** A cluster forming in the interior of an AD at P170. Arrows show the cluster border. **c** Yap1 is translocated to the nucleus in clusters. **d** The downstream target of Yap1, IL-6, is induced with nuclear translocation of Yap1 in clusters. **e** Cells in culture were allowed to reach confluence, and were then treated with 75 pM Tgf-β1 for 12 h. Note the block in Yap1 nuclear translocation at confluence. Similar cytoplasmic retention of Yap1 was evident at confluence in the absence of Tgf-β1. Zeb2 shRNA knockdown maintains nuclear Yap1 at confluence. **f** Real-time PCR showing Zeb1 mRNA is repressed and Zeb2 mRNA is induced when cells are allowed to become confluent. **g** Control cells remain growth-arrested at confluence, but knockdown of Zeb2 allows continued proliferation and cell outgrowth into foci. **h** Knockdown of Zeb1 leads to loss of proliferation and senescent morphology^29^. **i**–**i″** Yap1 is nuclear in subconfluent control cells. **j**–**j″** Yap1 becomes sequestered in the cytoplasm of subconfluent cells when Zeb1 is knocked down. Bars are 50 μm in panels **a**–**e**, and 25 μm in panels **f**–**j**

Nuclear Yap1 was not evident in AAH, adenomas, or surrounding normal lung, but nuclear translocation occurred specifically in hypoxic clusters where a Zeb2-to-Zeb1 switch occurs in a Tgf-β1-rich environment (Fig. 4a–c). IL-6 is a key downstream effector of Yap1 in progenitor cell expansion, and it is directly targeted by Yap1 for induction in vivo^44^. IL-6 marks sites of hypoxia in tumors, and its induction in early lesions is necessary for unrestricted cancer cell outgrowth^45^. Consistent with nuclear translocation of Yap1 in cell clusters, IL-6 induction was evident in these cells (Fig. 4d).

In cell culture, as reported previously^38^, we found that Yap1 became sequestered in the cytoplasm as cells reached confluence, and Tgf-β1 was unable to drive its nuclear translocation in these confluent cells (Fig. 4e). In these confluent cells, expression of Zeb1 mRNA was repressed and Zeb2 mRNA was induced (Fig. 4f). But, with Zeb2 knockdown, Yap1 persisted in the nucleus of confluent cells, and the cells lost contact inhibition allowing their outgrowth into foci (Fig. 4e, g). We conclude that Zeb2 is blocking nuclear translocation of Yap1. With Zeb1 knockdown, and the resulting elevation of Zeb2, Yap1 became sequestered in the cytoplasm of subconfluent cells, and these subconfluent cells stopped proliferation (Fig. 4h–j”). Taken together, our results suggest that Zeb1 repression of Zeb2 is a key focal point in the regulation of Tgf-β1 signaling, and nuclear translocation of Yap1 is linked to cell proliferation.

### Repression of Zeb2 Leads to Loss of Pten and Constitutive Akt Phosphorylation

**Fig. 5:**
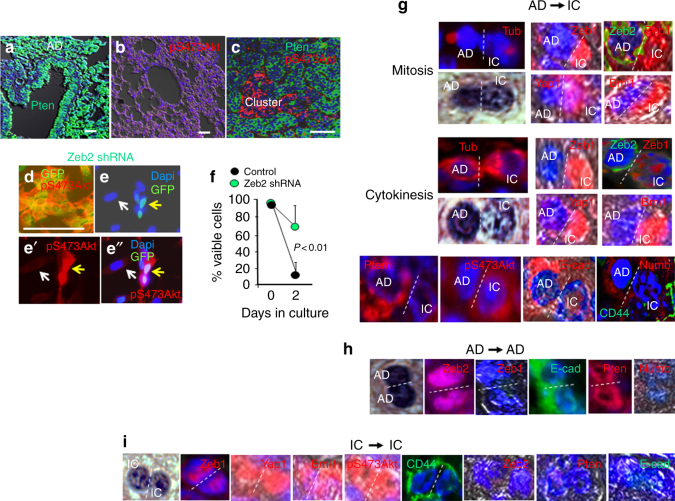
Pten loss and pS473Akt induction mark clusters, Zeb2 knockdown causes pS473Akt and resistance to anoikis, and asymmetric division of adenoma cells generates IC. **a** Immunostaining showing Pten in normal lung, AAH, and AD. **b** Immunostaining showing pS473Akt is low in normal lung, AAH, and AD. **c** Repression of Pten and induction of pS473Atk marks clusters. **d**–**e″** In cell culture, shRNA knockdown of Zeb2 causes constitutive pS473Akt. **d** shows a higher cell density region. GFP is expressed from the lentivirus. The control virus had no effect of pS473Akt. **f** Consistent with the anti-apoptotic properties of pS473Akt, Zeb2 knockdown cells, displaying constitutive pS473Akt, showed increased survival (resistance to anoikis) when placed in suspension culture for 48 h. Bars are 50 μm. **g** Dividing cells in clusters were identified by immunostaining for Tubulin (Tub)+ mitotic spindles. Cells displaying patterns of mitosis or cytokinesis, as well as the morphology of dividing cells, were examined. We noted mitotic cells where one pole displayed dense heterochromatin of AD cells, whereas the opposite pole showed decondensed chromatin of IC. Immunostaining showed that Zeb1, Yap1, and Bmi1 co-localized with decondensed chromatin, whereas Zeb2 was restricted to dense chromatin. This transcription factor polarization persisted in cytokinesis. The pole of the dividing cells with Zeb2+ compact chromatin showed an E-cadherin+, CD44−, Pten+, pS473Akt− pattern of parent adenoma cells during cytokinesis, whereas the opposite pole with Zeb1+, Bmi1+, Yap1+ decondensed chromatin had transitioned to an E-cadherin−, CD44+, pS473Akt+ pattern of daughter IC. Notably, Numb, which was not evident in adenoma cells, was induced in the dividing cells and it co-localized asymmetrically with E-cadherin during cytokinesis. **h** Outside of EF5+ regions in AD, AD cells divide symmetrically to produce AD daughters. **i** We identified other dividing cell in clusters that did not display nuclear polarization, and showed uniform chromatin decondensation in both poles. These dividing cells maintained the E-cadherin−, Zeb1+, CD44+, Zeb2−, Pten−, pS473Atk+, Bmi1+, nuclear Yap1 pattern of IC in both poles. Although Numb was maintained in these dividing IC, it was symmetrically distributed. Cells type and number of dividing cells in clusters is quantified in Fig. 1k

Zeb2 has been shown to act as a competitive endogenous RNA that sequesters miRNAs normally targeting Pten mRNA^33^. In this way, Zeb2 expression was shown to maintain Pten. Consistent with these findings, we found Pten diminished with Zeb2 repression in hypoxic clusters (Fig. 5a–c). In culture, *Zeb1* mutation or knockdown led to induction of Pten mRNA (Fig. 3c, e). Conversely, Zeb2 knockdown led to the loss of Pten mRNA (Fig. 3e). We then concluded that de-repression of Zeb2 following Zeb1 loss is responsible for this induction of Pten. Pten inhibits PI3K activity, preventing downstream PI3K-initiated mTocr2 activating phosphorylation of Akt on S473 (pS473Akt)^15^. Consistent with the loss of Pten in hypoxic clusters, pS473Akt accumulated in these cells (Fig. 5a–c). And, knockdown of Zeb2, leading to loss of Pten in culture, caused constitutive pS473Akt (Fig. 5d–e”). pS473Akt inhibits apoptosis, and it can prevent cell death when adhesion-dependent cells are placed in suspension culture (anoikis)^46^. Consistent with constitutive pS473Akt upon Zeb2 knockdown, these cells showed increased survival in suspension (Fig. 5f).

### Nuclear Polarization of Transcription Factors in Mitosis Establishes Parent and Daughter Fates Prior to Cytokinesis as Adenoma Cells Divide Asymmetrically to Generate IC

The appearance of IC in the interior of expanding adenomas suggested that they might be derived from adenoma cells. Dividing cells in hypoxic clusters at P170 were confirmed by Tubulin immunostaining of mitotic spindles, and cells displaying patterns of mitosis or cytokinesis were included (Fig. 5g). We identified mitotic cells where one forming pole displayed decondensed chromatin indicative of IC, whereas the opposite pole retained condensed chromatin of adenoma cells (Fig. 5g). In these mitotic cells, Zeb1, Bmi1, and Yap1 were restricted to decondensed chromatin, whereas Zeb2 was associated exclusively with compacted chromatin in the opposite pole. And, this polarization of the transcription factors persisted during cytokinesis (Fig. 5g). Consistent with this early nuclear polarization of transcription factors in mitosis driving gene expression patterns that distinguish parent adenoma cells and daughter IC, the pole of the dividing cells with Zeb2+ compact chromatin already showed an E-cadherin+, CD44−, Pten+, pS473Akt− pattern of parent adenoma cells during cytokinesis, whereas the opposite pole with Zeb1+, Bmi1+, Yap1+ decondensed chromatin had transitioned to an E-cadherin−, CD44+, pS473Akt+ pattern of daughter IC (Fig. 5g). We concluded that adenoma cells are dividing asymmetrically to generate IC, and nuclear polarization of transcription factors during mitosis is driving opposing target gene expression patterns, which define parent adenoma cells and daughter IC, in opposite poles of the cell before cell division is completed.

Numb is required for asymmetric division^47^. It binds to E-cadherin in the parental pole of asymmetrically dividing cells causing cytoskeletal rearrangements that drive unequal distribution of cellular content to daughter cells^48^. In this context, Numb blocks EMT linked to differentiation in the parental pole of asymmetrically dividing stem cells and CSC. Recent evidence demonstrates that asymmetric division is regulated by miRNAs targeting Numb mRNA, and miR-34a directly targets Numb mRNA, and repression of miR-34a by inflammation induces Numb to promote asymmetric division^49,50^. In addition to miR-200, Zeb1 represses miR-34a, and this loss of miR-34a was shown to drive cytoskeletal changes^51^. We did not detect Numb in Zeb1− adenoma cells, but it was induced with Zeb1 in mitotic adenoma cells giving rise to IC, and it co-localized asymmetrically with E-cadherin in the parental “adenoma-like” pole of these dividing cells (Fig. 5g, h).

As expected, adenoma cells distal to hypoxic clusters divided symmetrically to produce additional adenoma cells with the same nuclear morphology and expression pattern, consistent with the observed expansion of adenomas in the lung (Fig. 5h).

### IC in Clusters Divide Symmetrically to Expand Their Number

We identified other dividing cells in clusters at P170 that did not display nuclear polarization, and showed uniform chromatin decondensation of IC in both poles. Accordingly, these dividing cells maintained the E-cadherin−, Zeb1+, CD44+, Zeb2−, Pten−, pS473Atk+, Bmi1+, nuclear Yap1 pattern of IC in both poles (Fig. 5i). These results demonstrate symmetric division of IC to produce additional daughter IC accounting at least in part for the increase in cluster size from P150 to P170 (Fig. 1f, j, k).

### IC are Initial Cancer-generating Cells (CGC)

**Fig. 6:**
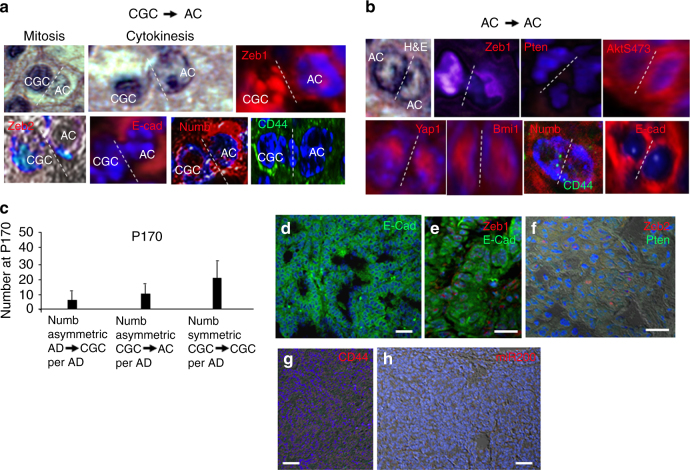
IC in clusters are cancer-generating cells (CGC). **a** In Fig. 5g, we identified AD cells dividing to produce IC daughters. We also noted a second form of nuclear polarization in dividing cells in clusters at P170. Partially decondensed chromatin indicative of IC was evident in one pole of these cells, whereas euchromatin indicative of cancer cells was present in the opposite pole. Zeb1 was enriched in decondensed chromatin, and this pole of the cell maintained the E-cadherin- and CD44^hi^ pattern of IC. The opposite pole of the cell with Zeb1^lo^ euchromatin displayed the E-Cadherin+ and CD44^lo^ pattern of cancer cells (AC), and Numb asymmetrically co-localized with E-cadherin. By contrast to Zeb1, Bmi1 and Yap1 were equally distributed in the decondensed chromatin and euchromatin of both poles, and both poles maintained a Zeb2−, Pten−, and pS473Akt+ pattern. We conclude parent IC are dividing to produce daughter AC, and they are therefore cancer-generating cells (CGC). **b** We identified symmetrically dividing cancer cells that displayed euchromatin as well as the same Zeb1^lo^, CD44^lo^, E-Cadherin+, Zeb2−, Pten−, pS473Akt+, Bmi1+, nuclear Yap1+ pattern in both poles. **c** Quantification of different cell divisions occurring in clusters. AD, CGC, and AC cells were identified in clusters by nuclear morphology and expression patterns. AD = Zeb2+, E-cad+; CGC = CD44^hi^; AC = Zeb2−, E-cad+. *n* = 10 clusters were averaged at P170. **d** Expanding tumors display papillary morphology and are E-cadherin (E-cad)+. **e** These E-cad+ tumors cells remain Zeb1^lo^. **f** Tumor cells are Pten− and Zeb2−. **g** CD44 expression is low on the tumor cells. **h** In situ hybridization shows that tumors cells are miR-200−. Bars are 50 μm

We then examined clusters for evidence that IC might be dividing to produce cancer cells. A second form of nuclear polarization was evident in dividing cells in clusters at P170. Partially decondensed chromatin representative of IC was evident in one pole of these cells in mitosis, whereas euchromatin indicative of cancer cells was present in the opposite pole (Fig. 6a). Zeb1 was enriched in decondensed chromatin, and this pole of the cell maintained the E-cadherin− and CD44^hi^ pattern of IC. The opposite pole of the cell with Zeb1^lo^ euchromatin displayed the E-Cadherin+ and CD44^lo^ pattern of cancer cells. By contrast to Zeb1, Bmi1 and Yap1 were equally distributed in the decondensed chromatin and euchromatin of both poles, and both poles maintained a Zeb2−, Pten−, pS473Akt+ pattern (Figs. 5g, h and 6a). Numb expression was asymmetric in these dividing cells, where it co-localized with E-cadherin in the pole displaying euchromatin (Fig. 6a, c). A Zeb1^lo^, CD44^lo^, E-Cadherin+, Zeb2−, Pten−, pS473Akt+, Bmi1+, nuclear Yap1+ pattern was maintained in cancer cells as they divided symmetrically to form papillary tumors (Fig. 6b, d–f). Numb expression was maintained in the cancer cells, but was equally distributed to both poles as these cells divided symmetrically (Fig. 6b, c). Consistent with ongoing repression of miR-200, the cancer cells were Bim1+, but they were CD44^lo^ (Fig. 6g, h). Thus, whereas ongoing repression of miR-200 in the cancer cells was sufficient for continued expression of Bmi1, it was not sufficient to maintain CD44^hi^. It then appeared a high level of Zeb1 might be necessary to maintain CD44 through a miR-200-independent pathway, or CD44 expression is reduced in cancer cells via a dominant Zeb1-independent mechanism (see below).

We concluded that IC divide asymmetrically to produce cancer cells, and they are thus initial CGC. Nuclear polarization of transcription factors again highlighted parent CGC and daughter cancer cells fates during mitosis. It then appears these CGC are a stable population bridging the gap between precancerous and malignant cells. Numb-highlighted asymmetric and symmetric divisions in clusters are quantified in Fig. 6c.

### Invading Cancer Cells Display Plastic EMT, but Fail to Induce CD44 or Display Properties of CGC

**Fig. 7:**
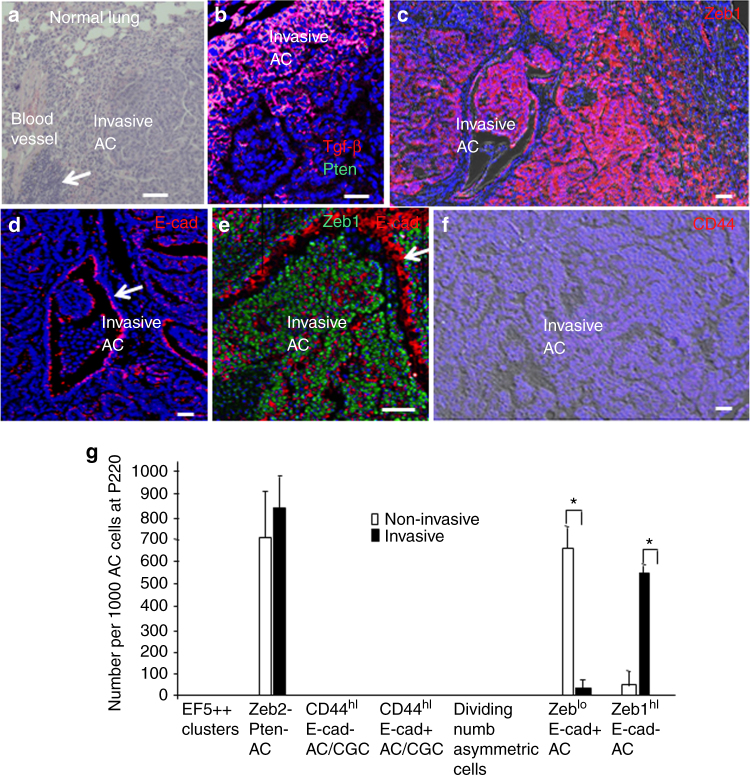
Invading cancer cells display plastic EMT, but fail to induce CD44 or show properties of CGC. **a** Sites of airway invasion show inflammation (arrow). **b** Sites of tumor invasion are Tgf-β1-rich and remain Pten−. **c** Zeb1 is re-induced in tumor cells at sites of airway invasion. **d** E-cadherin (E-cad) is repressed in tumor cells at sites of airway invasion, but it is maintained in airway epithelial cells (arrow). **e** Higher power view of Zeb1+, E-cad− tumor cells invading an airway. The arrow indicates E-cad+ airway epithelial cells. **f** Invading tumor cells continue to be CD44^lo^. **g** Quantification of cell morphology, expression, and division patterns in invasive and non-invasive tumors. *n* = 18 at P220. Bars are 50 μm

We reasoned one explanation for the Zeb1^lo^, CD44^lo^ pattern, and lack of EMT in the cancer cells (Fig. 6a–g) might be they are moving away from sites of hypoxia and Tgf-β1 accumulation as they expand into tumors. Notably, subsequent invasion of expanding tumors into airways occurs at sites of inflammation and Tgf-β1 accumulation (Fig. 7a,b), and we found that immunostaining for Zeb1 was increased in these invading cells and the cells had undergone EMT as evidenced by loss of E-cadherin (Fig. 7c–e). Based on our findings in cell culture, we suggest that ongoing repression of Zeb2 in the cancer cells allows the cells to respond to Tgf-β1 concentrated around airways and induce Zeb1 and EMT to facilitate invasion. However, despite this induction of Zeb1 and EMT and continued repression of miR200, the invading cancer cells remained CD44^lo^ (Fig. 7f), they did not show evidence of EF5++ clusters, a decondensed chromatin pattern resembling CGC, and we did not observe asymmetric distribution of Numb in any of the invading cells undergoing division (Fig. 7g). We conclude that EMT in cancer cells is plastic and linked to inflammatory infiltrate, Tgf-β1 accumulation, and resulting Zeb1 induction at sites of invasion. But, this induction of Zeb1 does not restore a CD44^hi^ CGC-like population among the invading cancer cells. Thus, it appears that the Zeb1/CD44 loop evident in CGC is disrupted by stable Zeb1-independent repression of CD44 that is initiated as cancer cells are generated.

### Zeb1 is Required for CGC Generation

We have shown that Zeb1 is important for tumor progression in K-Ras-initiated lung cancer^20^. But, the mechanism was unknown, and we concluded that the effects were likely due to a classic role for EMT in cancer cell behavior (e.g., migration and invasion). However, our results above raised the possibility that Zeb1 in the context of a CD44/Zeb1 loop might be critical for CGC formation and in turn cancer cell generation. Knockout of *Zeb1* leads to late embryonic lethality, and its loss causes changes in developing epithelial field borders^29,52^. Heterozygous mutation of *Zeb1* does not affect viability, but the mice develop posterior corneal dystrophy, demonstrating that simply lowering the level of Zeb1 is impactful in vivo^53^. And, we show here that heterozygous mutation or Zeb1 knockdown causes repression of CD44 and induction of Zeb2, suggesting this threshold level of reduction in Zeb1 is sufficient to deregulate genes marking the transition to CGC.

**Fig. 8:**
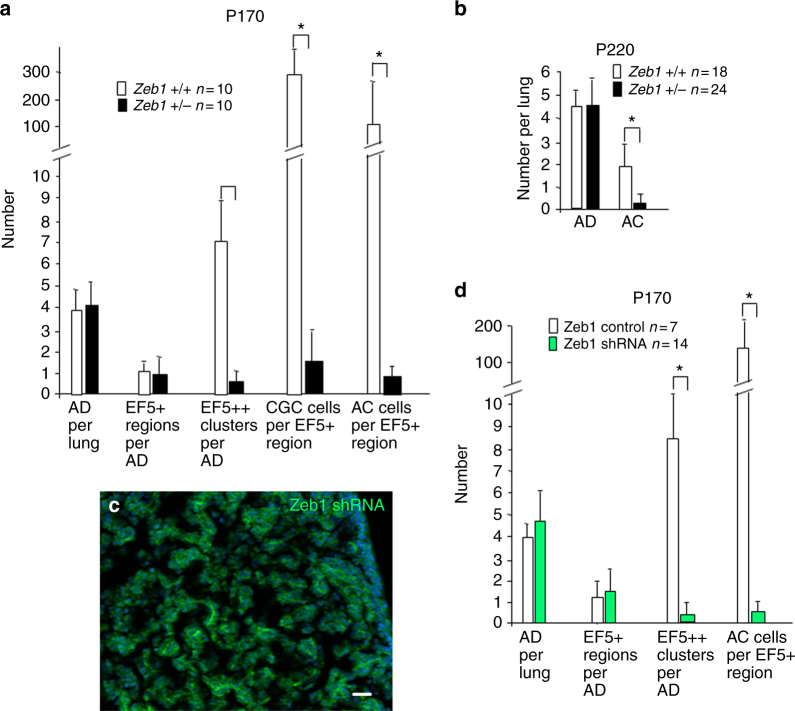
A threshold level of Zeb1 is required for CGC formation and cancer cell generation. **a** Quantification of the effects of heterozygous mutation of *Zeb1* on CGC at P170. EF5+ and EF5++ sites are described in Fig. 1d, e, k. CGC and AC cells in clusters were identified by nuclear morphology and expression patterns. CGC = CD44^hi^; AC = Zeb2−, E-cad+. **b** Heterozygous mutation of Zeb1 does not affect AD formation, but it inhibits AC. **c**, **d** Zeb1 shRNA or control lentivirus was delivered intratracheally biweekly beginning at P30 and ending at P90. Then, lungs were examined at P170. Panel **c** shows efficient infection of the lung assessed by GFP expressed from the virus. Quantification of the effect of the Zeb1 shRNA on EF5++ cluster formation and generation of AC cells in the EF5+ regions of AD is shown. EF5++ clusters and AC cells observed were GFP−, indicating they had arisen from uninfected cells. The bar is 50 μm

K-Ras mice were crossed into a *Zeb1*(+/−) background (Methods). The reduced level of Zeb1 had no effect on adenoma formation, or generation of hypoxia in interiors of expanding adenomas (EF5+) at P170 (Fig. 8a). But, small intensely EF5++ hypoxic sites indicative of cluster cells were inhibited, and accordingly, clusters of cells displaying decondensed chromatin failed to form (Fig. 8a). In turn, CD44 was not induced, Zeb2 was not repressed, and cells displaying an E-cadherin−, Pten− pattern of EMT were diminished in adenomas. Consistent with this inhibition in the formation of CGC, cancer cell number was inhibited as was AC outgrowth at P220 (Fig. 8a, b).

Lentivirus co-expressing GFP and Zeb1 shRNA, or a control sequence (Methods), was delivered intratracheally every 2 weeks into K-Ras mice beginning at P30 and ending at P90. Mice were then analyzed at P170, and using GFP as a marker, virus efficiently infected normal lung tissue (Fig. 8c). We did not detect a difference in GFP+ adenoma formation or transition of the interiors of these expanding adenomas to hypoxia (EF5+), but EF5++ clusters were inhibited as was cancer cell generation (Fig. 8d). We concluded that a threshold of Zeb1 induction is necessary to drive adenoma cells to produce CGC, and in the absence of CGC, initial cancer cells are not generated.

### Characterization of CD44^hi^ Cells from Lung Tumors

We attempted to isolate and compare adenoma, CD44^hi^ CGC, and cancer cells from K-Ras initiated tumors. First, we noted that neither adenoma cells at P150 nor cancer cells at P225 survived when lungs were dissociated and placed in cell culture. And, we found that CD44^hi^ cells in these primary cultures, or following sorting of dissociated lung tissue for CD44, rapidly lost CD44 in culture, and the cells failed to proliferate. p53 has been shown to repress CD44, and this repression of CD44 is important for p53 tumor suppression and cell growth arrest^11^. Progressive p53 induction in primary rodent cell culture is classically responsible for cell senescence. Even though p53 does not appear to affect cancer cell generation or proliferation in K-Ras tumors in vivo^18,19^, we hypothesized that its induction under the stress of culture was responsible for blocking CD44 expression and viability in culture.

**Fig. 9:**
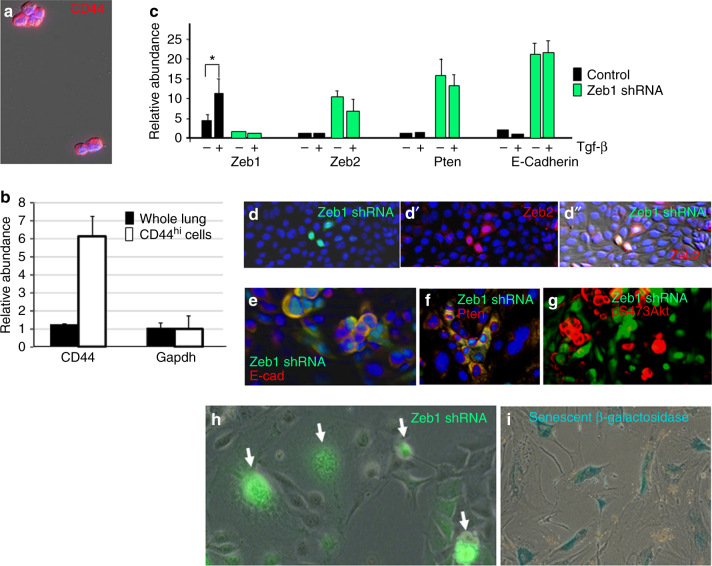
CD44^hi^ cells from K-Ras/p53 compound mutants. **a** Immunostaining demonstrating the viable cells from K-Ras, p53 mutant tumors are CD44^hi^. **b** Real-time PCR showing that the viable cells in culture are the CD44 mRNA high fraction of the lung tumors. **c** Real-time PCR showing that knockdown of Zeb1 in cells from **a** leads to induction of Zeb2 and Pten mRNAs, and with this increase in Zeb2, Tgf-β1 signaling is inhibited. **d**–**d″** Lentiviral infection with Zeb1 shRNA (expressing GFP) causes induction of Zeb2. **e** Knockdown of Zeb1 leads to induction of E-cadherin (E-cad). **f** With the increase in Zeb2 upon Zeb1 knockdown, Pten is induced. **g** Pten induction following Zeb1 knockdown leads to loss of constitutive pS473Akt. **h**, **i** Knockdown of Zeb1 leads to loss of cell viability. GFP+ cells show the large, flattened, and multi-nucleated morphology of senescent cells, and according the cells stained for senescent β-galactosidase, as described^29^

Notably, a small subset of cells was shown previously to expand when K-Ras, p53 compound mutant tumors were placed in culture, and these cells were highly tumorigenic when delivered intratracheally back into WT lungs^51^. Here, we show that this subset of cells surviving in culture from K-Ras, p53 compound mutant tumors are the CD44^hi^ population (Fig. 9a, b). As with CGC, these CD44^hi^ cells displayed a Zeb1+, Zeb2−, Pten−, pS473Atk+ pattern (Fig. 9c–g). Knockdown of Zeb1 lead to induction of Zeb2, and in turn, induction of Pten with loss of constitutive pS473Akt. This induction of Zeb2 also led to a block of Tgf-β1 signaling (Fig. 9c). Notably, knockdown of Zeb1 in these CD44^hi^ cells led to cell senescence (Fig. 9h, i), demonstrating they have become addicted to Zeb1. Taken together, these results suggest that p53 is a barrier to the culture of CD44^hi^ CGC, and, even with *p53* mutation, these CD44^hi^ cells represent the small population in the lungs that is viable in culture.

### A ZEB2 and PTEN Signature Predict Survival in Lung AC

**Fig. 10:**
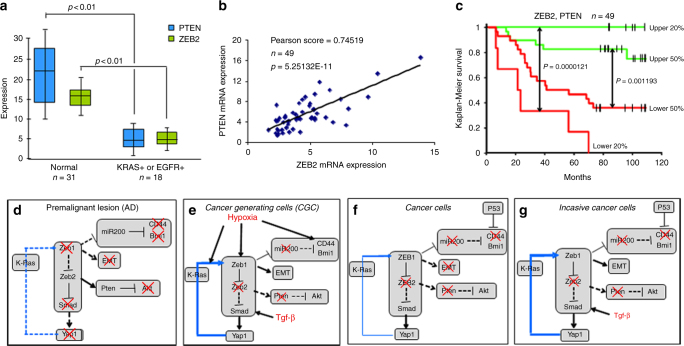
A ZEB2, PTEN predicts survival in human lung AC, and model of precancerous lesion transition to lung cancer. **a** Box plot showing expression of ZEB2 and PTEN mRNAs in human lung AC. The top of the box is the 75 percentile, whereas the bottom is the 25 percentile. The bar indicates the median and whiskers the highest and lowest values. K-Ras^+^ and EGFR^+^ indicate mutations confirmed by sequencing, and “Normal” indicates adjacent uninvolved lung tissue. **b** Pearson plot demonstrating a linear correlation between ZEB2 and PTEN mRNA levels in tumors in **a**. **c** Survival curves showing a ZEB2, PTEN expression signature predicts survival in human lung AC. **d** Precancerous adenoma (AD) lesions. K-Ras mutation triggers cell proliferation leading to the outgrowth of lung epithelial cells into AAH and AD. Red “X” indicates a block. The blue line running through K-Ras shows that, when activated, Yap1 can bypass the ongoing requirement for K-Ras mutation in tumors^40–42^. Dashed lines indicate inactivated pathways, whereas solid lines show activated pathways. The font size of Zeb1, Smad, and Yap1 depict relative level of expression or activation. **e** Cancer-generating cells (CGC). Hypoxia and Tgf-β1 accumulation in the interior of expanding AD triggers formation of CGC. **f** Cancer cells. As cancer cells form from CGC and migrate away from Tgf-β1-rich hypoxic clusters, Zeb1 expression diminishes. This lower level of Zeb1 is sufficient to maintain repression of miR-200 and induction of Bmi1, and repression of Zeb2 leading to repression of Pten and nuclear Yap1. But, this lower level of Zeb1 is not sufficient to maintain EMT. And, CD44 is low in the cancer cells, consistent with its dominant repression by p53. **g** Invasive cancer. As cancer cells encounter Tgf-β1-rich airways, Zeb1 is induced and the cells again undergo EMT. This depicts plastic EMT in cancer cells linked to invasion. But, this induction of Zeb1 in invading cancer cells does not lead to re-expression of CD44 or asymmetry of heterochromatin, Numb, and transcription factors in poles of dividing cells, as with CGC

Our results point to downregulation of Zeb2 as a key initiating event in loss of Pten expression and in unleashing Tgf-β1 signaling. This Zeb2−, Pten−, pattern established in cancer-generating cluster cells, persists in cancer cells. Notably, loss of Zeb2 provides a mechanism for Pten repression that is a hallmark of AC, where *Pten* is not mutated. In line with our findings in the mouse, both ZEB2 and PTEN mRNAs were downregulated in human lung AC with a Ras pathway mutation (Fig. 10a), and there was a linear correlation between their expression in the tumors (Fig. 10b), implying the same linkage in their expression we found in mouse lung tumors and cells in culture. Consistent with the importance of ZEB2 and PTEN, their co-expression was a remarkable predictor of patient survival (Fig. 10c).

## Discussion

Mutation of K-Ras leads to a prolonged period of precancerous adenoma outgrowth in the mouse lung, followed by cancer cell generation in the hypoxic, Tgf-β1-rich interiors of these expanding lesions (Fig. 10d–f). These cancer cells, in turn, proliferate into large AC that invade airways (Fig. 10g). We identify a two-step pathway where precancerous cells divide asymmetrically to produce an intermediate population of CGC, which in turn, divide asymmetrically to produce cancer cells. Studies of mammalian asymmetric division mechanism have largely focused on cell polarization causing the mitotic plane in driving cells to unequally partition cytoplasmic contents into daughter cells in a Numb- and Par-mediated process^54^. However, studies of asymmetric division in flies and worms have shown differences in heterochromatin on the two DNA strands arising during the S phase, linking fate of the two daughter cells to differential segregation of these sister chromatids based on heterochromatin content^55–57^. A DNA replication-mediate process highlighted by differential loading of new unmodified histones on leading vs. lagging DNA strands was proposed to drive the heterochromatin differences in sister chromatids, and these differences in histone modifications in turn allowed mother and daughter centrosomes to segregate sister chromatids based on heterochromatin content into the two daughter cells. The implication being that resulting differences in heterochromatin content would be expected to regulate differential transcription factor binding, gene expression, and thus phenotype in the daughter cells. Although our studies lack the mechanistic detail found to drive differential heterochromatin assembly on sister chromatids in asymmetrically dividing fly and worm stem cells, we provide consequential and complimentary evidence that similar asymmetric heterochromatin assembly in sister chromatids actually drives mitotic polarization of key transcription factors including Zeb1 in cancer cell initiation. And, we provide linked evidence that this mitotic polarization of transcription factors has already produced asymmetric expression of their key target genes in opposite poles of the cell before cytokinesis.

CGC arise at hypoxic sites in expanding adenomas and are marked by EMT and a CD44/Zeb1 loop. Disruption of this loop by mutation or knockdown of Zeb1 eliminates CGC formation from adenoma cells, thereby inhibiting cancer cell production. Notably, hypoxia induces expression of Zeb1^58^, and Zeb1 repressor activity and its function in EMT are mediated through interaction with the NADH-dependent co-repressor CtBP^59^. Hypoxia-driven aerobic glycolysis, leading to NADH generation, then allows CtBP, and in turn Zeb1, to act as an oxygen/metabolic sensor^60–62^. Zeb1 repression of Zeb2 in these cells leads to the repression of Pten expression that is a hallmark of K-Ras driven AC, and it releases Smad inhibition allowing Tgf-β1 signaling to drive nuclear translocation of the CSC transcription factor Yap1. Division of these CD44^hi^, Zeb1^hi^ cells generates CD44^lo^, Zeb1^lo^ cancer cells. The reduced level of Zeb1 in the cancer cells is not sufficient to maintain repression of E-cadherin, and EMT is reversed. However, the level of Zeb1 was still sufficient to maintain repression of Zeb2, allowing the cancer cells to respond to Tgf-β1 accumulating at sites of inflammation and airway invasion to re-establish Zeb1^hi^ and EMT in the invading cells. We suggest that this constitutive repression of Zeb2 leads to plastic EMT in the cancer cells dictated by inflammation and Tgf-β1 accumulation concentrated around airways.

Despite the restoration of a Zeb1^hi^ level in invading cancer cells, the cells remained CD44^lo^ and we failed to detect cells with properties of CGC within the tumors. We interpret these findings to mean that cells with properties of CSC are not being generated from existing cancer cells in this model. Notably, migrating cells with properties of CSC are also linked to metastasis, and despite the formation of large invasive AC, no metastasis was evident in this K-Ras model. As noted above, attempts to isolate CD44^hi^ CGC by flow or simply observing the cells after tumor dissociation in cell culture led to rapid loss of CD44 expression and failure of the cells to expand in culture. In contrast to the K-Ras model, a small subset of cells could be cultured from K-Ras, p53 compound mutant tumors, and these cells were highly tumorigenic when delivered intrathecally into the lung^51^. Here, we demonstrate these cells correspond to a CD44^hi^ subset of the tumors. p53 represses CD44, and we conclude that p53 induction in culture, which is classically responsible for senescence in primary rodent cell cultures, is likely causing the loss of CD44 expression and failure of expansion of the cells in culture. Evidence suggests that CSC in tumors are derived from existing cancer cells that undergo reversible p53-regulated reprogramming, and they are important for ongoing tumor expansion and tumor renewal following therapy. Indeed, p53 mutation is required for a CSC pool in mouse breast cancer^63^. p53 triggers stable repressive epigenetic changes^64^, and we suspect p53 in expanding cancer cells in the K-Ras model is responsible for maintaining CD44 silencing, even as Zeb1 is re-induced during the invasion (Fig. 10f, g). In this regard, Numb sustains high p53 activity^65^, and notably it was expressed in cancer cells, where it was symmetrically distributed along with E-cadherin in the dividing cells (Fig. 6b). We suggest this inability to induce CD44 in the cancer cells prevents their reprogramming to CSC until p53 is mutated. Although it remains to be seen how the CD44^hi^, Zeb1^hi^ CGC we identify as initial CGC within adenomas might be related to CSC, we propose CSC generation from existing cancer cells reflects reprogramming aimed at restoring the cancer cell-generating phenotype of CGC.

## Methods

### RNA Extraction and Real-time PCR

RNA was extracted using TRIzol, and cDNA was synthesized using the Invitrogen RT kit (Invitrogen), and SYBR Green real-time PCR was performed using a Stratagene Mx3000P Real-time PCR system^20^. PCR primer sequences and annealing conditions are shown in Supplementary Table 1. Three independent samples, each in triplicate, were analyzed for each real-time PCR condition. The detection of miRNAs was described previously^24^. Briefly, polyadenylation of at least 5 µg of total RNA was completed by poly(A) polymerase kit (PAP, Ambion) in 20 µl of reaction volume according to manufacturer’s instruction. The polyadenylated RNA was thereafter directly utilized for cDNA preparation using a reverse transcription kit (M-MLV reverse transcriptase, Invitrogen) and an adaptor primer (5′-GCGAGCACAGAATTAATACGACTCACTATAGG(T)12VN*-3′) in 40 µl of reaction volume. Real-time quantitative PCR was performed using a universal primer (5′-GCGAGCACAGAATTAATACGAC-3′) and a miRNA-specific primer (Supplementary Table 2). Tgf-β1 was from BioSource, Camarillo, CA.

### Immunostaining and In Situ Hybridization

Images were captured with a Zeiss Axio Imager.M2 microscope equipped with ApoTone.2 using a X-cite series 120Q light source, and AxioCam ICc3 and AxioCam MRm cameras. Images in the Zeiss Zen imaging program were transferred to Powerpoint for figure assembly. Only brightness and contrast were modified. Then a PDF was created. Immunostaining was performed as described previously^29^. Antibodies and conditions are described in Supplementary Table 3. EF5 staining for hypoxia was described previously^66^. For in situ hybridization, mouse lung tissues were fixed in 10% formalin solution immediately after removal, and then paraffin-embedded and sectioned at 10 µm. Locked nucleic acid-modified, double-digoxigenin (DIG)-labeled DNA probes were purchased from Exiqon (Denmark) and in situ hybridization was performed as we have described^67^. Briefly, the paraffin-embedded slides were deparaffinized and then treated with protease K. The probes were hybridized to the sections at 57 °C after dehydration, and then detected by an antibody against DIG conjugated with Cy3 (Jackson ImmunoResearch). miR-200 probes were described^67^.

### Chromatin Immunoprecipitation Assays

Primers, antibodies, and conditions for chromatin immunoprecipitation using Zeb1 and Zeb2 antibodies were described previously^20,67^.

### Cell Culture

Primary cultures of *Zeb1* mutant embryo fibroblasts were isolated as described^29^. Cells were cultured in Dulbecco's modified ealgle media (DMEM) with 10% heat-inactivated fetal bovine serum, or serum starved for 12 h prior to -β treatment. When lung tumors from K-Ras, p53 compound mutants were placed in culture, a small subset of these tumor cells survived and proliferated in culture, and these cells were highly tumorigenic when delivered intratracheally back into WT lungs^51^. These cells were grown in DMEM with 10% heat-inactivated fetal bovine serum. We found that this population was CD44^hi^.

### shRNA Knockdown

We have described lentiviral shRNA knockdown of Zeb1 and Zeb2 protein and mRNAs in detail previously^20,24,53,67^. Three shRNA lentiviruses with different Zeb1 and Zeb2 shRNA sequences from Open Biosystems were used for knockdowns with similar effects. Lentivirus with a scrambled shRNA sequence 5′-CAACAAGATGAAGAGCACCAATCTCTTGAAT TGGTGCTCTTCATCTTGTTG-3′ was used as a control—this control sequence was blasted against all mouse RNA sequences to ensure that it did not target an mRNA.

### Mutant Mice

Housing and handling of all mice were in accordance with procedures approved by the University of Louisville Institutional Animal Care and Use Committee (IACUC). K-ras^LA1^ mice^12^ in a C57BL/6 background were obtained from Jackson Laboratory. These mice were crossed with *Zeb1*(+/−) mice^29^ also in a C57BL/6 background to yield K-Ras^LA1^, *Zeb1*(+/+), and K-Ras^LA1^, *Zeb1*(+/−) mice. Lungs were obtained for histological analysis of tumors at the indicated ages. PCR genotyping was as described previously^20^. Tumor pathology was evaluated blindly by an experienced pathologist (M.C.). p53 mutant mice in a C57BL/6 background were also crossed to the latent K-Ras^LA1^ mice above and lung tumors were trypsinized and placed in cell culture in DMEM with 10% fetal bovine serum. A small fraction of cells survived, and RNA was isolated from these cells and starting lung tumor tissue for real-time PCR analysis of CD44 mRNA expression.

For lentiviral ZEB1 shRNA or control shRNA treatment, K-Ras^LA1^ mice at 1 month of age were anesthetized by IP injection of a combination of ketamine (100 mg/kg) and xylazine (10 mg/kg). The anesthetized mouse was placed on a warm platform in a biosafety hood. The mouth was opened by gently pulling the upper two front teeth, the tongue was extended by the forceps, and an Excel Saflet IV catheter was placed in the trachea. A total 50 µl of DMEM cell supernatant with a viral titer of approximately 1 × 10^9^ lentiviral particles was slowly delivered into the lung. The treated mice were then housed in a biosafety room, and closely monitored daily in accordance with protocols approved by the Institutional Animal Care and Use Committee. This tracheal viral delivery was repeated every 2 weeks until the animals were 3 months of age.

Animal number estimates were based on our previous studies using the K-Ras model^20^. Based on these previous studies, we did not detect any differences with regard to sex, thus male and female mice were chosen at random.

### Human Lung AC Microarray and Tissue Analysis

Microarray data for human lung AC sequenced for mutations in K-RAS and EGFR, and patient-matched control lung tissue was obtained from the NCBI database (GSE_11969). Data were corrected for background and normalized to median fluorescence and GAPDH expression. Box plots were done as described^33^.

### Statistical Analysis

Significance was determined by Student’s *t* test. Error bars in figures represent standard deviations. For survival curves, a logrank test was used to generate *p* values with the GraphPad Prism program.

### Data availability

All relevant data are available from the authors.

## Electronic supplementary material

Supplementary Information

## References

[1] Shibue T, Weinberg RA (2017). EMT, CSCs, and drug resistance: the mechanistic link and clinical implications. Nat. Rev. Clin. Oncol..

[2] Chaffer CL (2013). Poised chromatin at the ZEB1 promoter enables breast cancer cell plasticity and enhances tumorigenicity. Cell.

[3] Chaffer CL (2011). Normal and neoplastic nonstem cells can spontaneously convert to a stem-like state. Proc. Natl. Acad. Sci. U.S.A..

[4] Li R (2010). A mesenchymal-to-epithelial transition initiates and is required for the nuclear reprogramming of mouse fibroblasts. Cell Stem Cell.

[5] Wellner U (2009). The EMT-activator ZEB1 promotes tumorigenicity by repressing stemness-inhibiting microRNAs. Nat. Cell Biol..

[6] Wu YC, Tang SJ, Sun GH, Sun KH (2016). CXCR7 mediates TGFbeta1-promoted EMT and tumor-initiating features in lung cancer. Oncogene.

[7] Leung EL (2010). Non-small cell lung cancer cells expressing CD44 are enriched for stem cell-like properties. PLoS One.

[8] Brown RL (2011). CD44 splice isoform switching in human and mouse epithelium is essential for epithelial–mesenchymal transition and breast cancer progression. J. Clin. Invest..

[9] Xu H (2015). The role of CD44 in epithelial–mesenchymal transition and cancer development. Onco Targets Ther..

[10] Preca BT (2015). A self-enforcing CD44s/ZEB1 feedback loop maintains EMT and stemness properties in cancer cells. Int. J. Cancer.

[11] Godar S (2008). Growth-inhibitory and tumor-suppressive functions of p53 depend on its repression of CD44 expression. Cell.

[12] Johnson L (2001). Somatic activation of the K-ras oncogene causes early onset lung cancer in mice. Nature.

[13] Kwon MC, Berns A (2013). Mouse models for lung cancer. Mol. Oncol..

[14] Navas C (2012). EGF receptor signaling is essential for k-ras oncogene-driven pancreatic ductal adenocarcinoma. Cancer Cell.

[15] Song MS, Salmena L, Pandolfi PP (2012). The functions and regulation of the PTEN tumour suppressor. Nat. Rev. Mol. Cell Biol..

[16] Jin G (2010). PTEN mutations and relationship to EGFR, ERBB2, KRAS, and TP53 mutations in non-small cell lung cancers. Lung Cancer.

[17] Iwanaga K (2008). Pten inactivation accelerates oncogenic K-ras-initiated tumorigenesis in a mouse model of lung cancer. Cancer Res..

[18] Muzumdar MD (2016). Clonal dynamics following p53 loss of heterozygosity in Kras-driven cancers. Nat. Commun..

[19] Jackson EL (2005). The differential effects of mutant p53 alleles on advanced murine lung cancer. Cancer Res..

[20] Liu Y (2014). Different thresholds of ZEB1 are required for Ras-mediated tumour initiation and metastasis. Nat. Commun..

[21] Mainardi S (2014). Identification of cancer initiating cells in K-Ras driven lung adenocarcinoma. Proc. Natl. Acad. Sci. U.S.A..

[22] Sutherland KD (2014). Multiple cells-of-origin of mutant K-Ras-induced mouse lung adenocarcinoma. Proc. Natl. Acad. Sci. U.S.A..

[23] Liu C (2017). MicroRNA-141 suppresses prostate cancer stem cells and metastasis by targeting a cohort of pro-metastasis genes. Nat. Commun..

[24] Liu Y (2014). The ZEB1 transcription factor acts in a negative feedback loop with miR200 downstream of Ras and Rb1 to regulate Bmi1 expression. J. Biol. Chem..

[25] Gregory PA (2011). An autocrine TGF-beta/ZEB/miR-200 signaling network regulates establishment and maintenance of epithelial–mesenchymal transition. Mol. Biol. Cell.

[26] Lian I (2010). The role of YAP transcription coactivator in regulating stem cell self-renewal and differentiation. Genes Dev..

[27] Shimono Y (2009). Downregulation of miRNA-200c links breast cancer stem cells with normal stem cells. Cell.

[28] Crunkhorn S (2016). Cancer: BMI1 inhibition reverses lung cancer. Nat. Rev. Drug. Discov..

[29] Liu Y, El-Naggar S, Darling DS, Higashi Y, Dean DC (2008). Zeb1 links epithelial–mesenchymal transition and cellular senescence. Development.

[30] Shirakihara T, Saitoh M, Miyazono K (2007). Differential regulation of epithelial and mesenchymal markers by deltaEF1 proteins in epithelial mesenchymal transition induced by TGF-beta. Mol. Biol. Cell.

[31] Postigo AA, Dean DC (2000). Differential expression and function of members of the zfh-1 family of zinc finger/homeodomain repressors. Proc. Natl. Acad. Sci. U.S.A..

[32] Verschueren K (1999). SIP1, a novel zinc finger/homeodomain repressor, interacts with Smad proteins and binds to 5’-CACCT sequences in candidate target genes. J. Biol. Chem..

[33] Karreth FA (2011). In vivo identification of tumor-suppressive PTEN ceRNAs in an oncogenic BRAF-induced mouse model of melanoma. Cell.

[34] Caramel J (2013). A switch in the expression of embryonic EMT-inducers drives the development of malignant melanoma. Cancer Cell.

[35] Denecker G (2014). Identification of a ZEB2–MITF–ZEB1 transcriptional network that controls melanogenesis and melanoma progression. Cell Death Differ..

[36] Hegarty SV, Sullivan AM, O’Keeffe GW (2015). Zeb2: a multifunctional regulator of nervous system development. Prog. Neurobiol..

[37] Chng Z, Teo A, Pedersen RA, Vallier L (2010). SIP1 mediates cell-fate decisions between neuroectoderm and mesendoderm in human pluripotent stem cells. Cell Stem Cell.

[38] Hansen CG, Moroishi T, Guan KL (2015). YAP and TAZ: a nexus for Hippo signaling and beyond. Trends Cell Biol..

[39] Halder G, Dupont S, Piccolo S (2012). Transduction of mechanical and cytoskeletal cues by YAP and TAZ. Nat. Rev. Mol. Cell Biol..

[40] Zanconato F, Cordenonsi M, Piccolo S (2016). YAP/TAZ at the roots of cancer. Cancer Cell.

[41] Shao DD (2014). KRAS and YAP1 converge to regulate EMT and tumor survival. Cell.

[42] Kapoor A (2014). Yap1 activation enables bypass of oncogenic Kras addiction in pancreatic cancer. Cell.

[43] Pefani DE (2016). TGF-beta targets the Hippo pathway scaffold RASSF1A to facilitate YAP/SMAD2 nuclear translocation. Mol. Cell.

[44] Iliopoulos D, Hirsch HA, Wang G, Struhl K (2011). Inducible formation of breast cancer stem cells and their dynamic equilibrium with non-stem cancer cells via IL6 secretion. Proc. Natl. Acad. Sci. U.S.A..

[45] Kim T (2015). A basal-like breast cancer-specific role for SRF-IL6 in YAP-induced cancer stemness. Nat. Commun..

[46] Frisch SM, Screaton RA (2001). Anoikis mechanisms. Curr. Opin. Cell Biol..

[47] Bonifacino JS (2014). Adaptor proteins involved in polarized sorting. J. Cell Biol..

[48] Wang Z, Li SS (2010). Numb: a new player in EMT. Cell Adhes. Migr..

[49] Bu P (2013). A microRNA miR-34a-regulated bimodal switch targets Notch in colon cancer stem cells. Cell Stem Cell.

[50] Bu P (2016). A miR-34a-Numb feedforward loop triggered by inflammation regulates asymmetric stem cell division in intestine and colon cancer. Cell Stem Cell.

[51] Ahn YH (2012). ZEB1 drives prometastatic actin cytoskeletal remodeling by downregulating miR-34a expression. J. Clin. Invest..

[52] Takagi T, Moribe H, Kondoh H, Higashi Y (1998). DeltaEF1, a zinc finger and homeodomain transcription factor, is required for skeleton patterning in multiple lineages. Development.

[53] Liu Y (2008). Zeb1 mutant mice as a model of posterior corneal dystrophy. Invest. Ophthalmol. Vis. Sci..

[54] Neumuller RA, Knoblich JA (2009). Dividing cellular asymmetry: asymmetric cell division and its implications for stem cells and cancer. Genes Dev..

[55] Nakano S, Stillman B, Horvitz HR (2011). Replication-coupled chromatin assembly generates a neuronal bilateral asymmetry in *C. elegans*. Cell.

[56] Tran V, Lim C, Xie J, Chen X (2012). Asymmetric division of Drosophila male germline stem cell shows asymmetric histone distribution. Science.

[57] Xie J (2015). Histone H3 threonine phosphorylation regulates asymmetric histone inheritance in the Drosophila male germline. Cell.

[58] Zhang W (2015). HIF-1alpha promotes epithelial–mesenchymal transition and metastasis through direct regulation of ZEB1 in colorectal cancer. PLoS One.

[59] Postigo AA, Depp JL, Taylor JJ, Kroll KL (2003). Regulation of Smad signaling through a differential recruitment of coactivators and corepressors by ZEB proteins. EMBO J..

[60] Dias JM (2014). CtBPs sense microenvironmental oxygen levels to regulate neural stem cell state. Cell Rep..

[61] Shen Y (2017). Bioenergetic state regulates innate inflammatory responses through the transcriptional co-repressor CtBP. Nat. Commun..

[62] Di LJ (2013). Genome-wide profiles of CtBP link metabolism with genome stability and epithelial reprogramming in breast cancer. Nat. Commun..

[63] Chiche A (2017). p53 deficiency induces cancer stem cell pool expansion in a mouse model of triple-negative breast tumors. Oncogene.

[64] Kastenhuber ER, Lowe SW (2017). Putting p53 in context. Cell.

[65] Colaluca IN (2008). NUMB controls p53 tumour suppressor activity. Nature.

[66] Liu Y (2013). Repression of Zeb1 and hypoxia cause sequential mesenchymal-to-epithelial transition and induction of aid, Oct4, and Dnmt1, leading to immortalization and multipotential reprogramming of fibroblasts in spheres. Stem Cells.

[67] Liu Y (2013). Rb1 family mutation is sufficient for sarcoma initiation. Nat. Commun..

