# Supplementary material for: Mitotic polarization of transcription factors during asymmetric division establishes fate of forming cancer cells
Source: Nat Commun. 2018 Jun 21;9:2424. doi: 10.1038/s41467-018-04663-1 (PMC6013470; doi:10.1038/s41467-018-04663-1)
Supplement: Supplementary file 1 — Supplementary Information [file 41467_2018_4663_MOESM1_ESM.pdf]

Liu et al Supplemental tables

Supplementary table 1. Primers used for real-time PCR

Primer name	Sequence	Tm ˚C	Amplicon (bp)
Mm Pten LP	5'- TGGATTCGACTTAGACTTGACCT	55.3	182
Mm Pten RP	5'- TGGCGGTGTCATAATGTCTCT	56.0	
Mm Bmi1 LP	5'- ATCCCCACTTAATGTGTGTCCT	55.9	116
Mm Bmi1 RP	5'- CTTGCTGGTCTCCAAGTAACG	55.7	
Mm Zeb1 LP	5'- TGGCAAGACAACGTGAAAGA	60	200
Mm Zeb1 RP	5'- AACTGGGAAAATGCATCTGG	60	
Mm Zeb2 LP	5'- TAGCCGGTCCAGAAGAAATG	60.0	156
Mm Zeb2 RP	5'- GGCCATCTCTTTCCTCCAGT	61.0	
Mm ACTB LP	5'- GGCTGTATTCCCCTCCATCG	57.6	154
Mm ACTB RP	5'- CCAGTTGGTAACAATGCCATGT	55.9	

Supplementary table 2. Primers used for detecting miRs

Primer name	Sequence	Tm ˚C	Amplicon (bp)
Mm mir200c LP	5'- TAATACTGCCGGGTAATGATGGA	55.6	68
Mm mir200b LP	5'- TAATACTGCCTGGTAATGATGA	51	68
Mm mir200a LP	5'- TAACACTGTCTGGTAACGATGT	53.5	68
Mm miACTB LP	5'- GGCTGGCCTGTACACTGACTTGA	61.1	110
Mm miGAPDH LP	5'- CACTGAGCATCTCCCTCACA	56.6	155
Universal miR RP	5'- GCGAGCACAGAATTAATACGAC	53.9	

Supplementary table 3. Antibodies

Name	Cat#	Source	Host	Ab type	diluent
**Hypoxyprobe**	HP3-100 Kit	hpi			
**Akt / PKB[pS^{473}]**	44-621G	Invitrogen	Rb	Monoclonal	1:100
**Bmi-1, clone F6**	05-637	Millipore	mouse	Monoclonal	1:200
**(CD-44) HCAM (IM7)**	sc- 18849	Santa Cruz	Rat	Monoclonal	1:100
**E-Cadherin**	610181	BD Transduction Lab	mouse	Monoclonal	1:50
**IL-6 (M-19)**	sc-1265	Santa Cruz	Goat	Polyclonal	1:200
**PTEN(N-19)**	sc-6818	Santa Cruz	Goat	Polyclonal	1:50
**TGF-β1 (V)**	sc-146	Santa Cruz	Rabbit	Polyclonal	1:100
**YAP**	4912	Cell Signaling	Rabbit	Polyclonal	1:250
**Zeb1**	Liu et al 2008	Douglas S. Darling	Rb	Polyclonal	1:500
**SIP1(H-260) (ZEB2)**	sc-48789	Santa Cruz	Rb	Polyclonal	1:200
